# Enhancing Low-Light High-Dynamic-Range Image from Industrial Cameras Using Dynamic Weighting and Pyramid Fusion

**DOI:** 10.3390/s25082452

**Published:** 2025-04-13

**Authors:** Meihan Dong, Mengyang Chai, Yinnian Liu, Chengzhong Liu, Shibing Chu

**Affiliations:** 1 School of Physics and Electronic Engineering, Jiangsu University, Zhenjiang 212013, China; 2222226016@stmail.ujs.edu.cn (M.D.); 2222326012@stmail.ujs.edu.cn (C.L.); c@ujs.edu.cn (S.C.); 2 Nantong Yangtze Delta Academy of Intelligent Sensing, Nantong 226010, China; 3 State Key Laboratory of Infrared Physics, Shanghai Institute of Technical Physics, Chinese Academy of Sciences, Shanghai 200083, China; mengyang9999@126.com; 4 Jiangsu Engineering Research Center on Quantum Perception and Intelligent Detection of Agricultural Information, Zhenjiang 212013, China

**Keywords:** smart city surveillance, industrial camera, high-dynamic-range image enhancement, multi-scale image fusion, dynamic weight, pyramid fusion

## Abstract

In order to solve the problem of imaging quality of industrial cameras for low-light and large dynamic scenes in many fields, such as smart city and target recognition, this study focuses on overcoming two core challenges: first, the loss of image details due to the significant difference in light distribution in complex scenes, and second, the coexistence of dark and light areas under the constraints of the limited dynamic range of a camera. To this end, we propose a low-light high-dynamic-range image enhancement method based on dynamic weights and pyramid fusion. In order to verify the effectiveness of the method, experimental data covering full-time scenes are acquired based on an image acquisition platform built in the laboratory, and a comprehensive evaluation system combining subjective visual assessment and objective indicators is constructed. The experimental results show that, in a multi-temporal fusion task, this study’s method performs well in multiple key indicators such as information entropy (EN), average gradient (AG), edge intensity (EI), and spatial frequency (SF), making it especially suitable for imaging in low-light and high-dynamic-range environments. Specifically in localized low-light high-dynamic-range regions, compared with the best-performing comparison method, the information entropy indexes of this study’s method are improved by 4.88% and 6.09%, which fully verifies its advantages in detail restoration. The research results provide a technical solution with all-day adaptive capability for low-cost and lightweight surveillance equipment, such as intelligent transportation systems and remote sensing security systems, which has broad application prospects.

## 1. Introduction

**Figure 1:**
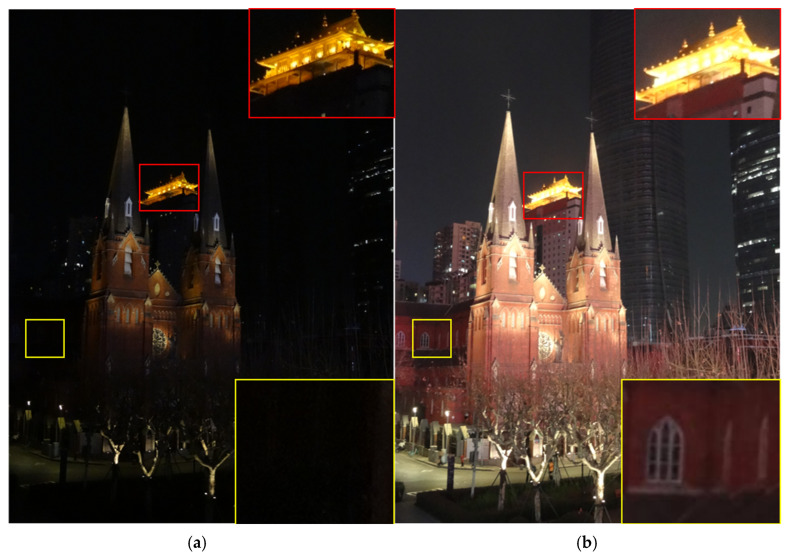
It is difficult for traditional equipment to simultaneously balance bright and dark areas in low-light high-dynamic-range scenes. (**a**) Underexposure of the dark areas (yellow boxes) when the bright areas are properly exposed; (**b**) overexposure of the bright areas (red boxes) when the dark areas are properly exposed.

With the modernization process of the city to update and promote, smart city security monitoring, intelligent transportation, and other areas that require target identification or monitoring of the field of all-day fine monitoring demand are increasingly becoming important [1,2,3]. In city streets, tunnels, and other complex lighting conditions, such as import and export monitoring, due to uneven and significant variations in lighting, it is difficult for traditional industrial equipment to simultaneously image “overexposed areas” and “underexposed areas” [2] (Figure 1), restricting the promotion of devices in refined applications. This is mainly because of the limited imaging capability of traditional devices [4], and the complex ambient lighting and changing weather conditions also present more demanding requirements for the adaptivity and robustness of algorithms [1,2].

In order to better cope with the imaging of such low-light high-dynamic-range scenes, researchers have proposed various schemes related to hardware and software. On the hardware side, the rapid development of high-dynamic-range imaging technology has enabled simultaneous improvement to the upper limit of pixel saturation [5] and low noise levels [6]; for example, Hsiu-Yu [5] and Arthur Spivak et al. [7] introduced the adaptive logarithmic response and pixel-by-pixel adaptive-exposure-time design on the pixel side of the detector, which realizes pixel-level dynamic range tuning in hardware. However, there are still issues such as the difficulty, complexity, and cost of hardware involved in simultaneously ensuring the quality of strong and weak signals in the image under extreme lighting conditions. On the software side, although many methods such as traditional histogram equalization [8], Retinex theory [9,10,11,12], image fusion [13,14,15,16], and deep learning [17] are able to equalize bright and dark regions to some extent and achieve better visual effects, they often produce halos in the processing of strong-light regions [18]. Deep learning methods rely on large-scale training data and hardware resources [19], which makes it difficult to satisfy the needs of low costs and miniaturization in the field of monitoring and control. Although the results of existing studies are good in some scenes, there is still a lack of complete solutions that can adapt to changing lighting environments [18] for the core objective of characterizing both light and dark textures under limited hardware conditions.

In this paper, we propose a new multi-exposure image fusion method based on the ratio pyramid and dynamic weights, which can balance bright and dark details in low- and medium-hardware configurations. Compared with the traditional Laplace pyramid fusion method, the ratio pyramid applies a multiplication strategy [20] in reconstruction; not only is this more advantageous in halo suppression, but also it better preserves dark details at night or in low-light environments. In the weight calculation, we incorporate the overall scene luminance information into the weight calculation so that the fusion result still has luminance balance and visual consistency in low-light high-dynamic-range scenes. In addition, the guided filter smoothing of the weight matrix can further suppress the noise and halo in the fusion process, ensuring that high stability and imaging quality are still maintained in scenes with large lighting differences. The experimental results show that this method outperforms existing multi-exposure fusion strategies in terms of dark detail enhancement, bright fidelity, and overall visual balance.

The rest of this paper is structured as follows: Section 2 reviews the related work, focusing on existing low-light enhancement methods and image fusion techniques. Section 3 details the proposed method for low-light high-dynamic-range image fusion. Section 4 verifies the effectiveness and adaptability of the proposed method through experimental analysis. Finally, the work of this paper is summarized, and an outlook is presented in Section 5.

## 2. Related Work

Currently, research on microlight image enhancement has made significant progress, which can be categorized into three main groups: traditional image enhancement methods, image fusion methods, and deep learning-based methods.

Early studies were mostly conducted from the perspective of image processing, such as histogram equalization (HE) and algorithms based on Retinex theory, which is commonly used for low-light enhancement and color correction by decomposing the light and reflectance components of an image in order to enhance contrast and eliminate the effects of non-uniform lighting. Pizer et al. (1987) [8] proposed a localized histogram equalization method, which enhances the local contrast of medical images. Rahman et al. (1996) [21] introduced multi-scale Retinex, which enhances image details while suppressing noise and has become an important theoretical cornerstone for subsequent studies. Subsequently, Rahman et al. (2004) [9], Setty et al. (2013) [10], and Gonzales et al. (2015) [11] made various improvements to the Retinex algorithm, which further enhanced the contrast and denoising ability of the image under low-light conditions. However, since most of these methods lack the full retention of edge information, when encountering low-light high-dynamic-range scenes where strong light and weak light coexist, halo amplification or overall overbrightness is prone to occur [12], which affects the visual quality of the image and the subsequent tasks, such as detection and recognition.

To better preserve details and balance bright and dark areas, multi-exposure or multi-sensor image fusion techniques [2] have received widespread attention. Yin et al. (2010) [22] fused infrared and visible images using the non-sampled contour wave transform and fuzzy logic to enhance visibility in low-light environments. Wang et al. (2011) [13], Singh et al. (2019) [14], Imran et al. (2019) [15], and Wu et al. (2023) [16] successively proposed the idea of image fusion based on a multi-scale pyramid. Among them, the pyramid decomposition method helps in detail enhancement and artifact suppression by decomposing the image at multiple levels in order to extract feature information at different scales. This method has been effective in reducing detail loss and artifacts in remote sensing and medical and consumer cameras. Compared with traditional enhancement methods, image fusion pays more attention to complementary information between different exposures or different source images, which can alleviate the problem of the co-existence of overexposure and underexposure to a certain extent. However, under complex lighting conditions, determining how to efficiently calculate the fusion weights and simultaneously suppress halo and noise amplification in strong light regions is still a direction worthy of further research [23]. In addition, multi-sensor image acquisition and alignment increase system cost and volume, which contradicts the lightweight requirement for practical security surveillance deployment [2].

In recent years, deep learning has shown great potential in the field of image enhancement. Zhang et al. (2019) [23] constructed a deep network named KinD based on Retinex theory; Lv et al. (2021) [24] introduced an attention mechanism in multibranch convolutional networks; and Chi et al. (2022) [25] designed a multi-scale feature fusion network with pyramidal attention, PAMF-NET, both of which achieved adaptive enhancement under low-light or complex lighting scenes and improved the detail expression and visual effect of images. Xing et al. (2023) [26] combined adaptive learning with convolutional neural networks and demonstrated better denoising and enhancement capabilities under complex lighting. However, deep learning methods rely on large-scale training data as well as hardware computational resources, and the complexity of the network structure also leads to high model deployment and update costs.

Among image fusion-based approaches, the Guided Filter-based Fusion (GFF) method [27] is widely recognized for its simplicity and efficiency, employing a guided filter to generate weight maps for multi-exposure or multi-scale fusion. Several studies show that GFF can effectively preserve edge details while smoothing noise in dimly lit scenes, yet its dynamic range expansion may be limited under highly uneven lighting, causing residual halo or over-smoothing in bright regions. Similarly, Li et al. introduced improved multi-scale decomposition and patch-based fusion strategies in 2017 (Li17) [28] and 2020 (Li20) [29]. The Li17 method used a Weighted Guided Image Filter to refine Gaussian pyramid weight maps, enabling more accurate structure-preserving fusion and better local brightness consistency, while the Li20 method further advanced fusion quality through fast multi-scale structural patch decomposition, mitigating ghosting and halo artifacts in dynamic scenes. Despite these refinements, both approaches can still face challenges with noise amplification in complex environments—e.g., Li17 may intensify unwanted artifacts in high-noise conditions, and Li20 can degrade near strong illumination boundaries. Although these fusion strategies generally outperform traditional Retinex-based methods in balancing bright and dark regions, their performance often depends on extensive parameter tuning and may falter under strong global illumination changes. These limitations motivate our pursuit of a more robust, lightweight solution for microlight image enhancement and fusion.

In addition to algorithmic advances, a range of metrics are used to evaluate low-light or high-dynamic-range image quality. In image fusion, these can be classified into four categories: (1) information-based (e.g., CE [30], EN [31], FMI [32], NMI [33], PSNR [34], *Q_NCIE_* [35]), (2) image-feature (e.g., AG [36], EI [37], *Q^AB/F^* [38], SD [39], SF [40]), (3) structural-similarity (e.g., *Q_Y_* [41], MEF-SSIM [42]), and (4) perception-inspired (e.g., *Q_cb_* [43], VIF [44]). Except for CE, higher values generally indicate better fusion performance. Key examples include EN for detail richness, AG and EI for edge and texture clarity, SD and SF for contrast and detail frequency, and PSNR or SSIM for fidelity and structural similarity. Perception-driven metrics such as *Q_cb_* or VIF capture subjective aspects of quality.

In summary, existing low-light high-dynamic-image enhancement techniques, whether based on traditional image processing, image fusion, or deep learning, have solved to some extent the problems of bright and dark areas not being able to be taken into account, details being easy to lose, and halo amplification, but practical applications still face the following two challenges: the limitations of computational and hardware resources, which need to be taken into account in the image enhancement effect under limited hardware costs, and adaptability to the changing lighting environment, where the lighting differences in different scenes and time periods require the method to have higher robustness and adaptability. Further research is needed on this matter.

## 3. Methods

### 3.1. Overview of the Methodology

**Figure 2:**
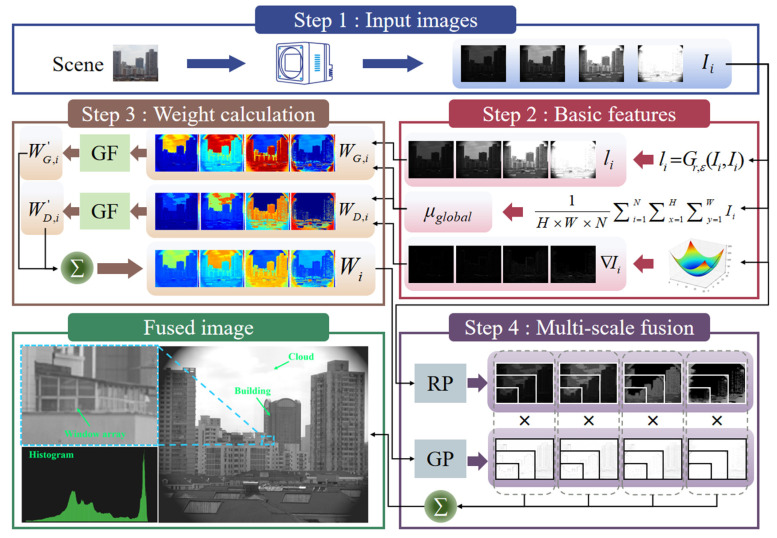
Schematic diagram of the multi-scale fusion framework proposed in this paper, which consists of input image sequences, basic feature computation, global/detail weight matrix computation, and pyramid decomposition and fusion, totaling four key steps.

In order to realize the acquisition of high-quality images under multiple high-dynamic-range lighting environments, such as daytime, nighttime, and strong and low light, we adopted a multi-scale fusion framework based on the pyramid structure (Figure 2) to simultaneously integrate the bright and dark details in a multi-frame image sequence. The method does not require the construction of complex physical imaging models and does not rely on high computational resources, which provides good practicality and robustness. The core idea of the method is to decompose the image in the multi-scale space; combine the global and local brightness, overall gradient, and other features to dynamically compute the weight matrix and smooth it; and ultimately fuse it layer by layer to generate a high-fidelity enhanced image.

As can be seen from Figure 2, in a high-dynamic-range scene, the images obtained by general imaging devices are either under- or overexposed. After the fusion of these differently exposed images using the present method, different light and dark details in the same image are better rendered. Traditional multi-scale fusion methods usually use pyramid decomposition, where the image is decomposed into sub-bands of multiple scales and the coefficients of each scale are weighted and fused to reconstruct the image. Such methods mainly rely on fixed weight calculation strategies, e.g., based on features such as contrast, gradient, or entropy, and although they can enhance details, they may suffer from insufficient luminance equalization and halo amplification in low-light and high-dynamic-range scenes.

In this study, we address these issues and propose an improved multi-scale fusion strategy to better equalize the light and dark information and enhance the adaptability to multiple lighting conditions. We first extract features such as global mean luminance ($\mu_{global}$), local luminance ($l_{i}$), and overall gradient ($\nabla I_{i}$) from the input image sequence. The global average luminance ($\mu_{global}$) is used to measure the overall luminance distribution of the whole frame to distinguish between scenes that are too dark or too bright; the local luminance ($l_{i}$) is obtained by the guided filter and smoothing operation, which can reflect the luminance difference of different regions; and the overall gradient ($\nabla I_{i}$) is used to obtain the edge and texture information of the image, thus reflecting the distribution of the details of the image. Based on these features, the global weight ($W_{G,i}(x,y)$) and detail weight ($W_{D,i}(x,y)$) of each image are calculated, and the guided filter (GF) is applied to smooth them in order to suppress the halo that is prone to appear in the fusion process. Finally, the fused images are generated by pyramid decomposition and multi-scale weighted fusion, in which the fusion results are gradually superimposed from the low to the high level to maximize the preservation of high-frequency texture information.

The specific formulas and principles used in the methodology of this study are described in detail below.

### 3.2. Low-Light Image Enhancement Method Based on Dynamic Weighting and Pyramid Fusion

In low-light and high-dynamic-range scenes, it is difficult to achieve a better visualization of images with complex lighting conditions using a single exposure function or a fixed luminance reference [45]. Images with low overall luminance are highly susceptible to loss of details due to the neglect of dark region weights in conventional fusion methods, which significantly affects image quality and usability [29]. In the light–dark junction regions, the unsmoothness of the weighting transition triggers glare spill and halo effects, which make the articulation of these regions unnatural, and even triggers the white light phenomenon at the edges [18]. In addition, significant changes in scene brightness at different times and lighting conditions, such as glare during daytime, low light at night, and low brightness distribution in shadows, further increase the difficulty of image processing based on the fixed-weight approach [2] and presents higher requirements on the adaptability of fusion algorithms. To address these issues, there is an urgent need to design a fusion strategy that can dynamically adjust the weights to better balance the local and global brightness, retain rich details, and ensure the overall consistency of the visual effect.

For this reason, we split the fusion weights into two parts: global weights, which are used to control the overall exposure level, and detail weights, which are used to enhance the local structure and texture. For the halo problem in the bright light region, this method combines guided filtering to smooth the weights and ensure that the fusion process still has a smooth transition and structural consistency at the edges with high contrast.

#### 3.2.1. Basic Feature Extraction from Images

(a) Smoothing and localized brightness

Smooth convolution is performed on each input image $I_{i}\in R^{H\times W\times N}$ to obtain the initial noise reduction$\;L_{i}$. Subsequently, local brightness $l_{i}$ is computed by guide filtering to balance noise suppression and edge preservation, as follows:

(1) $$l_{i}=G_{r,\varepsilon }(I_{i},I_{i})$$

Here, we use $G_{r,\varepsilon }(X,Y)$ for the guided filter operator, r for the filter radius, and ε for the regularization parameter to control the degree of blurring. *x* and *y* represent the input image and bootstrap image, respectively.

(b) Global average brightness

In scenes with a wide range of luminance variations, the exposure mismatch problem is prone to occur if we only rely on fixed exposure or local luminance information. In order to realize the dynamic adjustment of the overall exposure, we additionally calculate the global average luminance of all images $\mu_{global}$ and use it as an important factor for adaptive weighting at each stage, as follows:

(2) $$\mu_{global}=\frac{1}{H\times W\times N}\sum_{i=1}^{N}\sum_{x=1}^{H}\sum_{y=1}^{W}I_{i}(x,y)$$

where *N* is the number of images, and *H* and *W* denote the image height and width, respectively. Dynamic adjustment based on global brightness can accurately adjust the weighting ratio when overall darkness or overexposure occurs, which reduces the exposure mismatch problem due to the fixed exposure strategy compared with the traditional method that only sets a fixed exposure reference value.

(c) Gradient magnitude

High-dynamic-range scenes in low-light environments usually have a lot of critical structural information in the edge region. However, conventional fusion or noise reduction processes often result in blurred edges or missing high-frequency details. For this reason, we introduce a gradient magnitude $\nabla I_{i}(x)$ into the design of the fusion weights to highlight the detailed regions in the image, as follows:

(3) $$\nabla I_{i}(x,y)=\sqrt{{\left(\frac{\partial I_{i}}{\partial x}\right)}^{2}+{\left(\frac{\partial I_{i}}{\partial y}\right)}^{2}}$$

By calculating the gradient magnitude of each pixel point, high-frequency and structurally distinct regions can be given higher weights to enhance the preservation of key contours and textures in the final fusion. Compared with the traditional method of assigning weights relying only on brightness or contrast, the gradient magnitude can accurately perceive the edge regions, effectively inhibit the edge blurring problem that may occur when multiple images are superimposed, and enhance the clarity and structural integrity of the fusion result.

#### 3.2.2. Global Weight Calculation

In scenes with high dynamic range or an extremely uneven luminance distribution, if we only rely on a fixed reference value to measure the exposure degree, it is easy to see a clear deviation. For example, the method based on the traditional exposure function with a fixed reference value of 0.5 in the literature [45] cannot be flexibly adjusted according to the overall lightness or darkness of the actual scene brightness, as follows:

(4) $$E(x,y)=\exp\left(-\frac{(I(x,y)-0.5{)}^{2}}{2\sigma^{2}}\right)$$

To address this limitation, we propose global weights $W_{G,i}(x,y)$ combining the global average luminance $\mu_{global}$ with the local luminance $l_{i}$, which realizes the dynamic correction of the reference value when the light distribution is extremely uneven, so the image sequences can obtain a more appropriate weight assignment to the actual situation, as follows:

(5) $$W_{G,i}(x,y)=\exp\left(-0.5\times \left(\frac{{\left(\bar{I_{i}}-\mu_{global}\right)}^{2}}{\sigma_{g}^{2}}+\frac{{\left(l_{i}(x)-\mu_{global}\right)}^{2}}{\sigma_{l}^{2}}\right)\right)$$

where $\bar{I_{i}}$ denotes the average luminance of the ith image, and $\sigma_{g}$ and $\sigma_{l}$ control the sensitivity of the global and local luminance difference, respectively. When the overall or local brightness of an image is close to $\mu_{global}$, the corresponding region will obtain a higher weight; if the gap with this value is larger, the weight will be smaller.

Compared with the traditional method that only relies on a fixed reference value of 0.5, our method can adaptively increase the dark weights in low-light environments to ensure that more dark details are preserved; at the same time, it can also provide a certain degree of suppression of the strong-light areas in overbright scenes to avoid overexposure and loss of details. This comprehensive consideration of global and local brightness effectively solves the exposure mismatch problem caused by purely relying on fixed exposure values and provides more stable performance when dealing with complex and dynamic lighting environments.

#### 3.2.3. Detail Weight Calculation

In low-light or high-dynamic-range environments, high-frequency information such as edges and textures are extremely important. To further highlight the key structural features, we define the detail weight matrix${\;W}_{D,i}(x,y)$. Based on [28], we additionally introduce a gradient magnitude factor to more accurately characterize the edge strength, as follows:

(6) $$W_{D,i}(x,y)=\exp\left(-\frac{{\left(L_{i}(x,y)-\mu_{global}\right)}^{2}}{D^{2}}\right)\times f\left(\nabla I_{i}(x,y)\right)$$

where *D* is the detail preservation parameter and $\nabla I_{i}(x)$ is the pixel gradient magnitude.

Compared with the traditional fusion strategy that relies only on luminance or contrast, we incorporate the gradient magnitude into the weight calculation so that high-gradient regions (edges, texture-rich places) receive higher weights in the final fusion, avoiding dark edges from being drowned by noise or being over-smoothed in the low-light scene. At the same time, very dark regions with flat luminance or noise dominance have smaller gradients, and the corresponding weights are weakened, reducing possible artifacts and over-enhancement problems. As a result, our method is able to strike a better balance between noise suppression and detail preservation, which is particularly suitable for complex environments such as low-light and nighttime high-dynamic-range imaging.

#### 3.2.4. Guided Filter Smoothing

In high-contrast areas, too drastic a weight jump can cause halos or white edges. To suppress this phenomenon, guide filtering is applied to $W_{G,i}(x,y)$ and${\;W}_{D,i}(x,y)$, as follows:

(7) $$W_{G,i}^{\prime }=G_{r,\varepsilon }(W_{G,i},I_{i})\;W_{D,i}^{\prime }=G_{r,\varepsilon }(W_{D,i},I_{i})$$

The operators of guide filtering have been mentioned previously, except that the input here is the weight matrix, and the guide image is the original input image itself. In this case,${\;W}_{G,i}^{\prime }(x,y)$ and${\;W}_{D,i}^{\prime }(x,y)$ represent the global weights and detail weights after guide filtering, respectively. This operation effectively smooths the abrupt changes in light and dark edges and suppresses the appearance of halos while keeping the weights consistent with the image structure.

#### 3.2.5. Final Weighted Weight Matrix

The final weighted weight matrix is obtained by combining the global and detail weights. These two weights are aggregated by means of a weighted sum [46], as follows:

(8) $$W_{i}(x,y)=\alpha W_{G,i}^{\prime }(x,y)+W_{D,i}^{\prime }(x,y)$$

where α is the luminance factor to control the contribution of the global weights. The global weights contribute to the overall brightness balance, while the detail weights ensure that the fine structure is preserved. The combined weight matrix is subsequently normalized to ensure that the sum of the weights of all the input images at each pixel point is equal to 1, thus maintaining the consistency of the overall brightness.

#### 3.2.6. Multi-Scale Integration

After obtaining the final weighted weight matrix, the Gaussian pyramid and ratio pyramid are used to fuse the light and dark details of multi-frame images. Since pyramid decomposition has been widely used in the field of image fusion [20], this section only briefly describes the key processes involved.

First, let $GP_{0}$ denote the original input image and use it as the 0th layer of the Gaussian pyramid, and then the kth layer of the Gaussian pyramid is

(9) $$GP_{k}(i,j)=\sum_{m=-2}^{m=2}\sum_{n=-2}^{n=2}\omega (m,n)GP_{k-1}(2i+m,2j+n)$$

where ω(m,n) is a Gaussian window function with low-pass characteristics.

Second, in order to make the size of $GP_{k}^{*}$ the same as that of $GP_{k-1}$, the Gaussian pyramid $GP_{k}$ is enlarged by using interpolation to obtain the enlarged image $GP_{k}^{*}$, $GP_{k+1}^{*}$ is used as the background image of $GP_{k}$, and then the ratio pyramid of the image is

(10) $$\left\{\begin{array}{c} RP_{k}=\frac{GP_{k}}{GP_{k+1}^{*}},0\leq k\;<\;N\\ RP_{N}=GP_{N},k=N \end{array}\right.$$

where the highest level of the ratio pyramid is the *N*th level and $RP_{K}$ denotes the image of the *k*th level of the ratio pyramid.

Finally, as in [28], the number of layers of the pyramid is set. For each scale of the pyramid, the fusion process is as follows:

(11) $$R_{l}(x)=\sum_{k}GP_{l}\left\{W_{k}^{\left(i\right)}(x)\right\}RP_{l}\{I_{g,k}^{\left(i\right)}(x)\}$$

where *l* denotes the number of pyramid layers,$\;GP_{l}\left\{W_{k}(x)\right\}$ denotes the Gaussian pyramid decomposition of the final weight matrix, and$\;RP_{l}\{I_{k}(x)\}$ denotes the ratio pyramid decomposition of a sequence of multi-exposure input images. This results in a fused image, as follows:

(12) $$R_{f}(x)=\sum_{l}Up\left(R_{l}(x)\right)$$

where Up(⋅) is a continuous upsampling symbol with coefficients of $2^{l-1}$.

This process enables the separate treatment of light and dark parts in multi-scale space, avoiding over- or under-enhancement in single-scale enhancement approaches [2].

### 3.3. Summary

In this study, our method introduces the dynamic calculation of global average brightness and gradient magnitude enhancement and combines guided filtering to smooth the two types of weights, ultimately completing the image fusion under the multi-scale pyramid framework. From a macroscopic perspective, the global weights can dynamically assign exposure weights for a wide range of lighting differences, adapting to all-day scenes; from a microscopic perspective, the detail weights can ensure that dark or high-frequency structures can be emphasized, and the visibility of shimmering and complex texture regions can be significantly enhanced. The use of guided filtering effectively suppresses the halo caused by the strong contrast between light and dark parts, smoothing the fusion result in the transition between light and dark. Subsequent experiments will further verify the robustness and advantages of this method under various lighting environments.

### 3.4. Dataset and Experimental Environment

**Figure 3:**
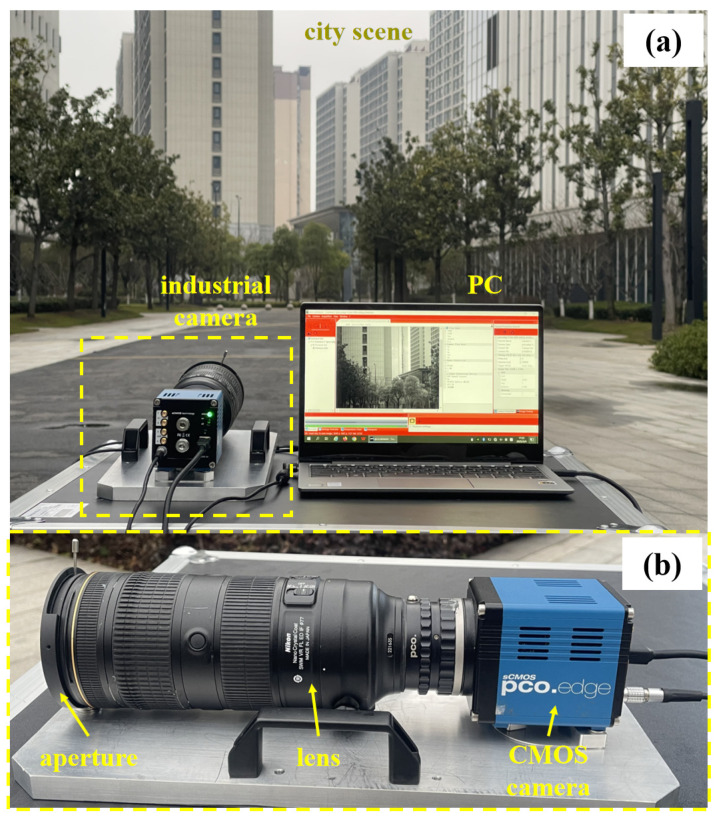
Complete image of our system: (**a**) our system; (**b**) a detailed introduction to the industrial camera we use.

In this study, we used a pco.edge 5.5 sCMOS camera, which was made in Germany, with a Nikon AF-S 70–200 mm F2.8E lens made in Japan, to acquire image sequences of different exposures under 24 h light conditions. The image sequences were taken from 6:00 a.m. on 28 September 2024, to 5:00 a.m. on 29 September 2024, in Shanghai, China, at 121.49 degrees east longitude and 31.26 degrees north latitude. The acquired images had a resolution of 2152 × 2560 and covered light scenes ranging from low light at night to high light during the day. The exposure time was adaptively selected based on the average pixel value of the scene, and the exposure aperture was manually adjusted according to the exposure level of the scene. All the experiments were realized on a PC equipped with an Intel(R) Core(TM) i5-8265U CPU @1.60 GHz. A complete image of our system is shown in Figure 3.

In order to comprehensively verify the advantages of the methods in this study, three representative image fusion methods were selected for comparison: guided-filtering-based image fusion (GFF) [27], multi-scale fusion based on detail enhancement (Li17) [28], and multi-exposure image fusion based on fast multi-scale structural block decomposition (Li20) [29]. The methods were also evaluated both qualitatively and quantitatively.

For the evaluation metrics, in order to measure the performance of the fused images in terms of information content, edge clarity, luminance level, texture richness, and visual coherence, we chose six metrics: information entropy (EN) [31], average gradient (AG) [36], edge intensity (EI) [37], standard deviation (SD) [39], spatial frequency (SF) [40], and the human visual perception metric (*Q_cb_*) [43]. Larger values of all metrics indicate better performance. The following text will further introduce the calculation methods of each evaluation index. In the following definitions, M and N represent the width and height of the image, respectively, while A and B represent two source images (i.e., image A and image B). Here, two input images are taken as examples for explanation. F represents the fused image.

(a) Information Entropy (EN) [31]

The formula for calculating information entropy (EN) is

(13) $$EN=-\sum_{l=0}^{L-1}p_{l}\log_{2}p_{l}$$

where *L* is the number of gray levels, and *p_l_* is the normalized histogram of the corresponding gray levels in the fused image. Information entropy obtains the amount of information contained in the fused image.

(b) Average Gradient (AG) [36]

The average gradient (AG) reflects the details and texture of the fused image by calculating the gradient information of the fused image. The calculation method is:

(14) $$AG=\frac{1}{MN}\sum_{i=1}^{M}\sum_{j=1}^{N}\sqrt{\frac{\nabla F_{x}^{2}(i,j)+\nabla F_{y}^{2}(i,j)}{2}}$$

where:

(15) $$\nabla F_{x}(i,j)=F(i,j)-F(i+1,j)$$

(16) $$\nabla F_{y}(i,j)=F(i,j)-F(i,j+1)$$

(c) Edge Intensity (EI) [37]

Edge intensity (EI) is used to measure the edge intensity information of an image. It can use the Sobel operator to calculate

(17) $$EI=\sqrt{S_{x}^{2}+S_{y}^{2}}$$

where

(18) $$S_{x}=F*h_{x}$$

(19) $$S_{y}=F*h_{y}$$

The symbol * represents the convolution operation.

(d) Standard Deviation (SD) [39]

The calculation method for standard deviation (SD) is

(20) $$SD=\sqrt{\sum_{i=1}^{M}\sum_{j=1}^{N}{\left(F(i,j)-\mu \right)}^{2}}$$

Among them, µ represents the average value of the fused image.

(e) Spatial Frequency (SF) [40]

The calculation method for spatial frequency (SF) is

(21) $$SF=\sqrt{RF^{2}+CF^{2}}$$

where

(22) $$RF=\sqrt{\sum_{i=1}^{M}\sum_{j=1}^{N}{\left(F(i,j)-F(i,j-1)\right)}^{2}}$$

(23) $$CF=\sqrt{\sum_{i=1}^{M}\sum_{j=1}^{N}{\left(F(i,j)-F(i-1,j)\right)}^{2}}$$

which is capable of calculating the gradient distribution of an image.

(f) Human Visual Perception Metric (*Q_cb_*) [43]

The calculation method for human visual perception (*Q_cb_*) is

(24) $$Q_{cb}=\frac{1}{MN}\left(\sum_{i=1}^{N}\sum_{j=1}^{M}\lambda_{A}(i,j)Q_{A,F}(i,j)+\lambda_{B}(i,j)Q_{B,F}(i,j)\right)$$

Among them, *Q_A,F_(i,j)* and *Q_B,F_(i,j)* represent the contrast from the source image to the fused image, and *λ_A_* and *λ_B_* represent the saliency maps of *Q_A,F_(i,j) and Q_B,F_(i,j)*, respectively. *Q_cb_* mainly reflects the similarity of the main features in the human visual system.

More information on various evaluation indicators can be obtained from the relevant literature, and will not be elaborated here.

We used two sets of parameter settings in a moderately illuminated scene and a low-light scene: 1.5 for α, 0.3 for $\sigma_{g}$, 0.3 for $\sigma_{l}$, and 40 for *D* for the moderately illuminated background, and 1 for α, 0.8 for $\sigma_{g}$, 0.8 for $\sigma_{l}$, and 3.6 for *D* for the fused low-light background.

## 4. Experimental Results and Discussion

### 4.1. Comparison of Overall Effect at 24 h

**Figure 4:**
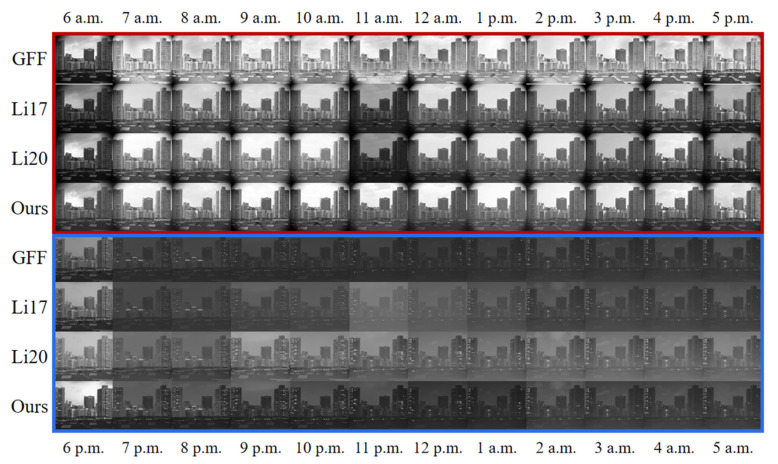
Overall comparison of the processing results of the GFF [27] method, the Li17 [28] method, the Li20 [29] method, and the method developed in this study, at different time points, in order to observe the transitions of the different methods under different lighting conditions. In the figure, the red box indicates the effect of the time period when light is still available, and the blue box indicates the effect of the time period when the light is faint.

For all-day light conditions, the processing results of the four methods are first qualitatively compared (Figure 4), covering early morning, midday, evening, and nighttime. As a whole, the GFF [27] method is prone to produce halos at the junction of buildings and the sky; the Li17 [28] method makes the overall brightness darker in backlit scenes, such as 11:00 a.m. and other periods with large light contrast; and at night, there is an inconsistent transition between dark and light in the overall picture due to the change in cloud thickness (11:00 p.m., 12:00 p.m.). The results of the Li20 [29] method is similar to that of the Li17 [28] method. The effect of the Li20 [29] method during daytime hours is similar to that of Li17 [28], with a slight lack of brightness transition in the picture, but during nighttime shimmering hours, it has better brightness and contrast, but still, in the scene with LED light strips (7:00 p.m., 8:00 p.m.), the image is darker than that in the neighboring time period. By contrast, this study’s method can sufficiently transition the overall brightness of each time period during daytime, without darkening when backlighting, and without a clear halo problem; at night, when the strong light and the dark part coexist, it can also keep the transition reasonable and natural, and there is no abnormal transition of the image brightness caused by the environment change, which demonstrates the strong adaptability and robustness of this study’s method.

**Figure 5:**
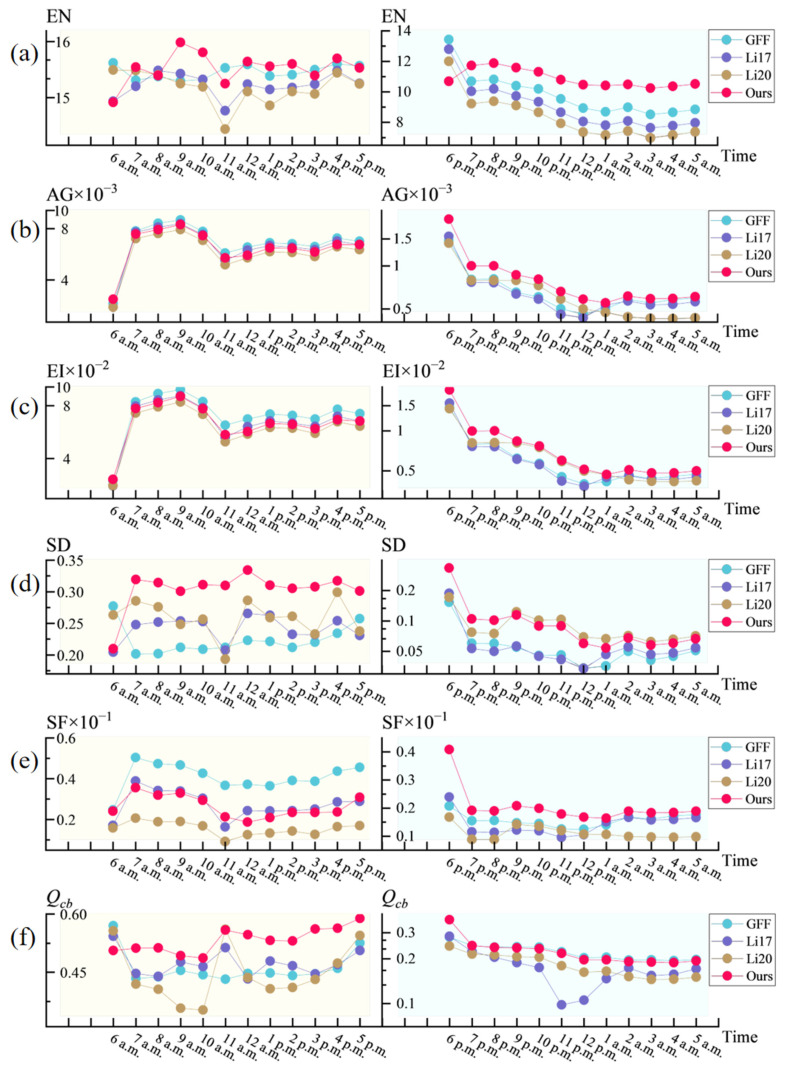
Line chart of the situation of the six evaluation indicators at 24 h for each method. Higher is better for all metrics. (**a**) EN, (**b**) AG, (**c**) EI, (**d**) SD, (**e**) SF, (**f**) *Q_cb_*.

In terms of quantitative analysis, we use six evaluation metrics to compare the performances of the four methods under all hours of the day and night. Among them, the information entropy (EN) [31] reflects the amount of information and the richness of details in an image; the average gradient (AG) [36] and the edge intensity (EI) [37] measure the structural clarity and the sharpness of the edges of an image, respectively; the standard deviation (SD) [39] and the spatial frequency (SF) [40] measure the breadth of the luminance distribution and the richness of the texture of an image, respectively; and the human visual perception metric (*Q_cb_*) [43] approximately simulates the human eye’s perception of the overall visual consistency of an image. The evaluation indicator line chart in Figure 5 shows the performances of the six indicators for the four methods in a 24 h scenario. For ease of observation, we divided the data into two groups based on time points, daytime and nighttime time periods, and presented them in some line graphs using logarithmic axes. Among them, the red part represents the data of this article.

Under daylight conditions, both the GFF method and our approach achieve high EN values (exceeding 15), indicating sufficiently rich information content. However, under low-light conditions, our approach exhibits significantly higher EN values, improving by 9.76% to 20.28% over the GFF method. This result demonstrates the superiority of our method in preserving texture and details under low-light scenarios. Regarding the AG and EI metrics, our method also outperforms other methods in low-light environments, with increases of approximately 2% to 23.56% over the second-best method, reflecting its advantage in maintaining detail sharpness. For the SD metric, our method shows a more pronounced advantage under daylight conditions but is slightly inferior to Li20 in low-light environments. This is because the guided filter employed in our method smooths the weights at the expense of some brightness contrast. With respect to the SF metric, GFF excels during daytime, while our method achieves the best performance under low-light conditions, outperforming the second-best method by about 7.09% to 41.94%. The *Q_cb_* metric indicates that our approach provides higher visual evaluation in daylight scenarios, while its performance in low-light scenarios is comparable to that of GFF, with differences ranging from 0.07% to 4.05%.

Overall, our method stands out across different time periods, matching or exceeding the best results among the alternative methods in certain scenarios.

### 4.2. Comparative Analysis of Daytime Scenes

**Figure 6:**
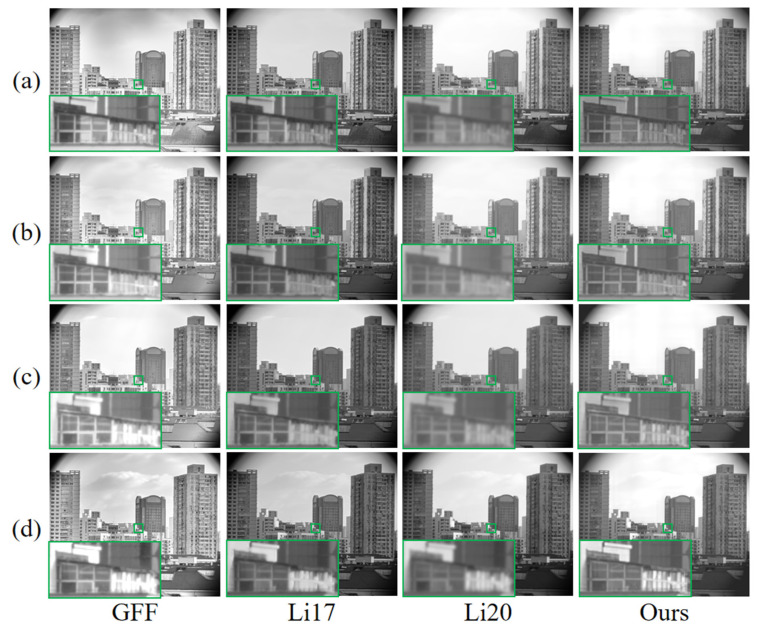
Detailed rendering of a scene during the hours with better light. Among them, the green boxes are used to facilitate comparison in detail. Scenes taken at (**a**) 7:00 a.m., (**b**) 10:00 a.m., (**c**) 1:00 p.m., and (**d**) 4:00 p.m.

**Figure 7:**
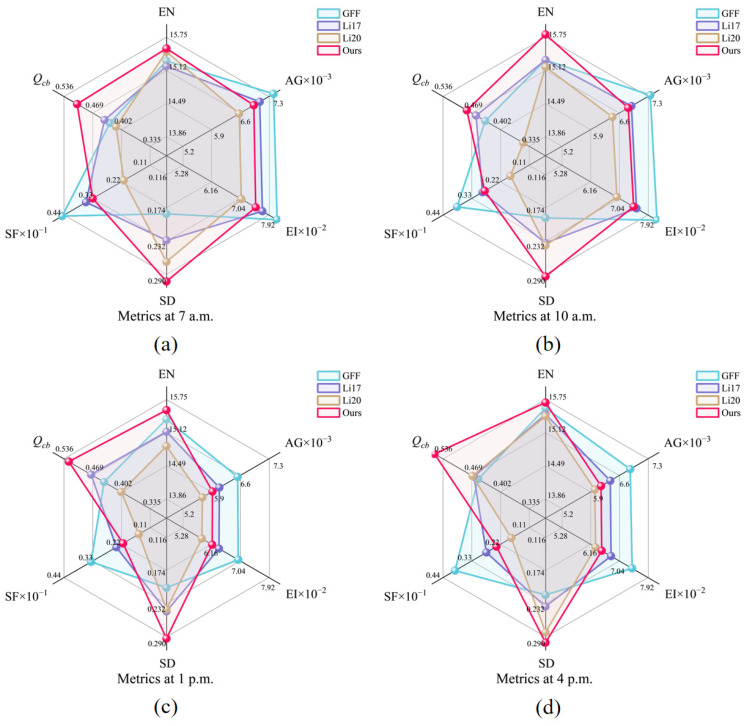
Radar plots comparing the metrics of different methods at four time points when the lighting is better: (**a**) 7:00 a.m., (**b**) 10:00 a.m., (**c**) 1:00 p.m., and (**d**) 4:00 p.m.

To further examine the effect of detail rendering in better daytime lighting, Figure 6 selects four scenes at 7:00 a.m., 10:00 a.m., 1:00 p.m., and 4:00 p.m. for local zoom-in, and compares the evaluation metrics of these four sets of scenes with the radargram (Figure 7). GFF and Li17 provide stronger edge sharpening in some areas, so the AG, EI, and SF metrics are generally high, but there is also slight haloing or over-enhancement. Li20 has a softer brightness overall, but detail-rich areas such as window frames are not finely processed, resulting in the blurring of detail magnification. Our method deals with the transition between strong light and shadow in a more balanced way, avoiding the loss of details caused by the bright and dark intersections. As seen in Figure 7, this method is superior in EN, SD, *Q_cb_*, etc., during daytime, which indicates that it is more outstanding in information richness, luminance hierarchy, and visual consistency; however, due to the introduction of bootstrap filtering, part of the high-frequency sharpening effect is sacrificed, which results in AG and EI being slightly inferior to the comparison method that focuses on strengthening the gradient. Overall, it seems that each method can have some effect in the application of daytime scenes.

In order to further quantify the comprehensive performance of each index, this study weights the six dimensional indexes one by one after scaling them to the [0, 1] interval equally according to the maximum value, and obtains the final comprehensive score. The upper limit of the score is set to 6 due to the number of indexes. The scoring results for the above scenarios are shown in Table 1: both this study’s method and the GFF method can obtain a high evaluation (more than 5 points), and the gap between them is in the range of −4.57% to +0.53%. (A positive value indicates that our method’s score exceeds the GFF method’s score by that percentage, whereas a negative value indicates that it is lower by that percentage.) These results suggest that, under well-lit daytime conditions, both methods achieve satisfactory visualization performance. As lighting conditions deteriorate, the difference in scores between our method and the GFF method diminishes, indicating that our method is particularly well suited for low-light scenarios. While there is some discrepancy in the scores of the two methods, the difference remains relatively small and is unlikely to significantly affect real-world application performance.

### 4.3. Comparative Analysis of Low-Light Scenes

**Figure 8:**
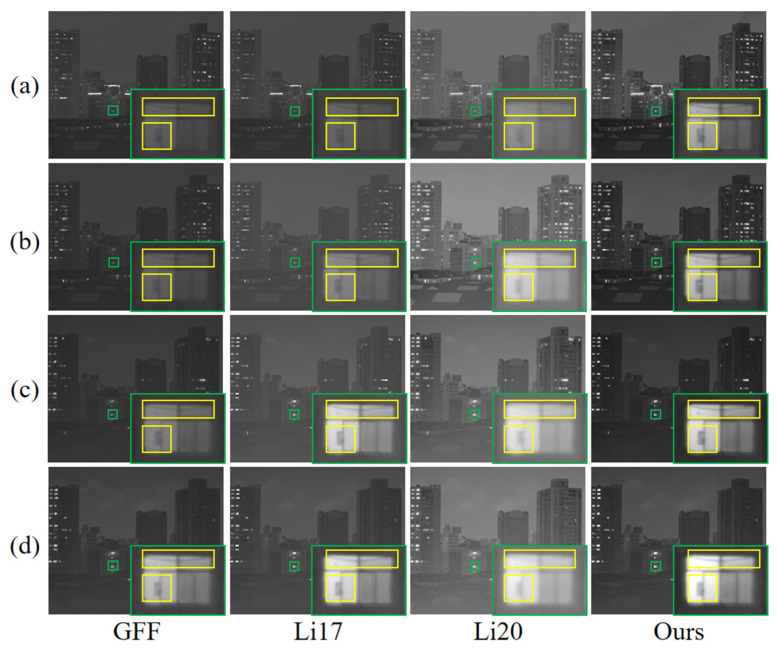
Effect of detail rendering of windows during the low-light time period. Among them, the green boxes are used to facilitate comparison in detail. The yellow boxes emphasize more detailed contrast. Time point scenes at (**a**) 7:00 p.m., (**b**) 10:00 p.m., (**c**) 1:00 a.m., and (**d**) 4:00 a.m.

**Figure 9:**
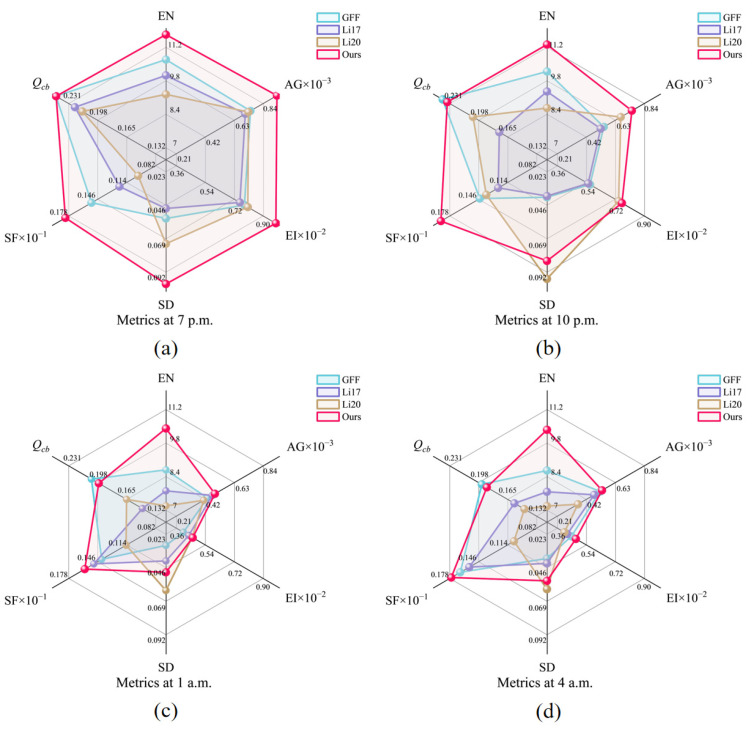
Radar plots comparing metrics of different methods at four time points during the low-light hours: (**a**) 7:00 p.m., (**b**) 10:00 p.m., (**c**) 1:00 a.m., and (**d**) 4:00 a.m.

Nighttime low-light and high-dynamic-range scenes place a higher demand on the robustness of the fusion algorithm. Figure 8 shows the local detail magnification comparison of four nighttime periods, 7:00 p.m., 10:00 p.m., 1:00 a.m., and 4:00 a.m., where the 7:00 p.m. scene is a low-light and high-dynamic-range scene, and the illumination of the 10:00 p.m., 1:00 a.m., and 4:00 a.m. scenes is much more subdued. From the detailed zoomed-in image of the window, the resultant image processed by this study’s method shows clearer lines and textures inside the yellow frame and higher contrast between light and dark inside the window, this study’s method effectively avoids overexposure through multi-scale adaptive weighting and guided filter smoothing, and the dark details are still well preserved. The radar image (Figure 9) further supports this point, and this method is superior in EN, AG, EI, SF, etc., especially in the high-dynamic-range environments at 7:00 p.m. Almost all the indexes are higher than that of the comparison method. However, the values of SD and *Q_cb_* are slightly lower than those of GFF and Li20 in very dark scenes, which is due to the fact that the guided filtering partially smoothens the parts with luminance differences, but in low-light scenes, this step does not significantly affect the overall sharpness and detail definition of the image. Overall, the present method is significantly better than the other methods in more complex and significant shimmering scenes.

The composite scores for the above night scenes are shown in Table 2: the method of this study achieves excellent results in all groups of scenes, especially in the 7:00 p.m. low-light high-dynamic-range scene, and the score is as high as 5.9993, which is almost the full score of 6. The scores of the remaining three scenes are also more than 5.7, and the composite scores are improved by 9.57–15.29% compared with that of the second-ranked method. The results fully reflect the comprehensive advantages of this study’s method in low-light environments, providing more robust and feasible imaging quality for practical applications in night scenes.

### 4.4. Comparative Analysis of Low-Light High-Dynamic-Range Scenes

**Figure 10:**
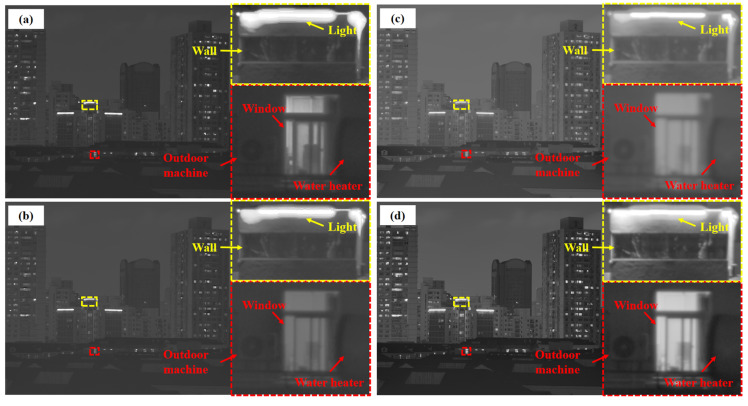
Localized high-dynamic-range display plots for each method in 8:00 p.m. scene. The yellow boxes show the wall and LED regions, and the red boxes show the window regions. (**a**) GFF, (**b**) Li17, (**c**) Li20, and (**d**) ours.

To better demonstrate the advantages of the proposed method in low-light, high-dynamic range scenes, select 8:00 p.m. for comparative analysis (Figure 10), which exhibits significant lighting differences. Especially the part of the Lighting Emitting Diode (LED) light strip, this study’s method did not show the halo problem, blurring problem, and weak contrast problem that appeared in the other methods in terms of halo processing of the image, enhancement in texture, and the contrast of the image as a whole in the two points of time. The present method gives a good rendering of the entire scene within the display frame, including the LED lights, the contents of the pictures on the wall, the windows, the heater, and the air-conditioning unit.

**Figure 11:**
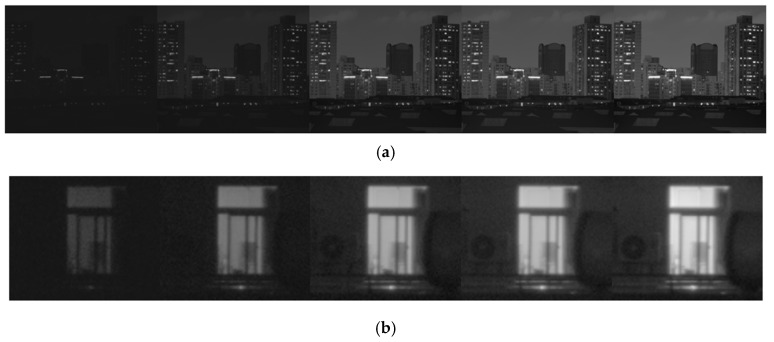
Multi-exposure image sequences of 8:00 p.m. scene captured by camera: (**a**) original image and (**b**) local high-dynamic-range window area in the original image.

From a visual standpoint (Figure 11), the window interior rendered by the GFF method often appears clearer than our approach. However, after repeated comparisons with the original multi-exposure sequence (Figure 11), we find that GFF tends to over-enhance high-frequency details in high-dynamic-range scenes, particularly where steep gradients are present. This leads to amplified contrasts and halo artifacts, resulting in certain areas becoming overly pronounced. By contrast, our method only applies guided filtering to in assist weight calculation and mitigate halo effects, thus avoiding similar over-enhancement.

**Figure 12:**
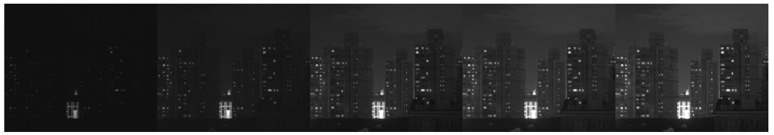
The original sequence of multi-exposure images captured by camera in new urban scene.

**Figure 13:**
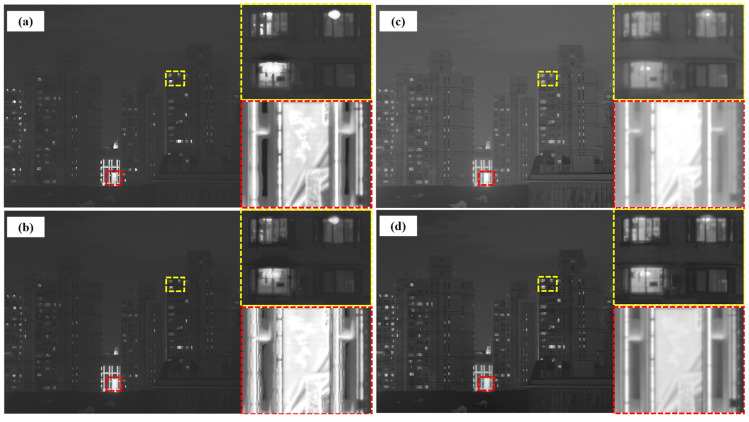
New urban scene: comparison of the effects of four methods and display of local high-dynamic-range effects. The yellow boxes show the window regions, and the red boxes show the LED regions. (**a**) GFF, (**b**) Li17, (**c**) Li20, and (**d**) ours.

**Figure 14:**
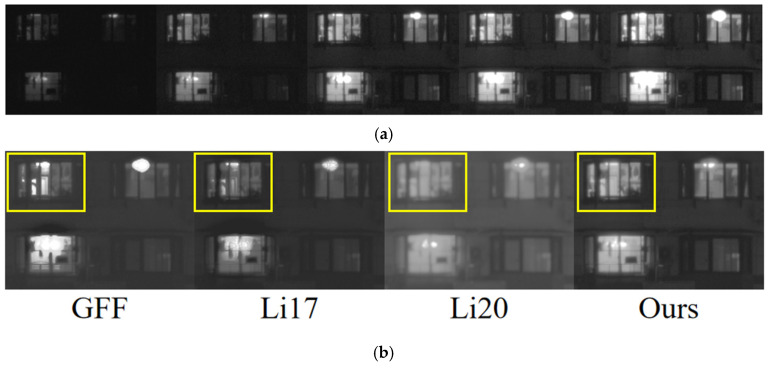
New urban scene: (**a**) original image of multi-exposure sequence of window area captured by camera; (**b**) comparison of window areas processed by various methods. The yellow boxes emphasize a more detailed contrast.

To further validate this observation, we introduced a new urban scene containing window and LED regions (Figure 12 and Figure 13) and compared the fused window areas with their counterparts in the original sequence (Figure 14). Combined with our daytime scenario (Figure 6), these results consistently confirm GFF’s tendency toward over-enhancement under strongly contrasting conditions. Nonetheless, GFF demonstrates impressive performance in moderate or daylight scenes, achieving crisp details and favorable contrast in many cases.

**Figure 15:**
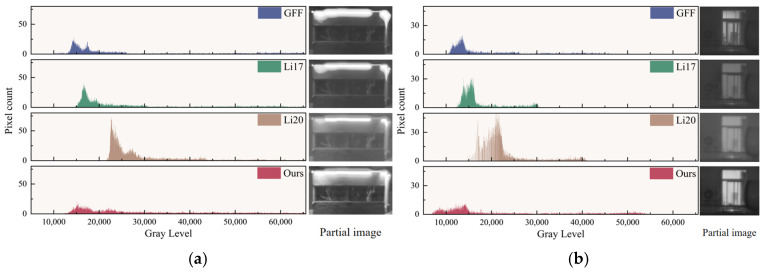
Histogram comparison of localized zoomed-in plots at 8:00 p.m. for (**a**) walls and LED strips and (**b**) windows.

We then analyze the gray-level histograms (Figure 15, Table 3) of two localized areas—one containing a wall and LED strip and another focusing on the window region—both captured at 8:00 p.m. Across GFF, Li17, Li20, and our method, the proposed approach achieves the highest overall dynamic range. In the wall/LED region, our gray-level standard deviation reaches 13,572, only 0.8% below GFF’s 13,683, indicating a comparable contrast. However, in the window region, our SD increases to 13,617, about 78.6% higher than GFF’s 7625, signifying a substantial advantage in differentiating bright and dark details.

Peak frequency also reveals how methods distribute gray values. In the wall/LED area, GFF attains the lowest peak frequency (29) among other competing methods, while ours is 20; similarly, in the window region, GFF’s peak frequency is 20 versus our 12. This disparity indicates that the other methods’ gray values cluster around narrower luminance ranges, whereas our approach maintains a more balanced distribution and thus a richer dynamic range.

Finally, we examine information entropy, which quantifies the amount of detail in the fused image. Our method achieves the highest entropy scores among all four methods—12.50 in the wall/LED region (4.88% above the best comparison) and 11.32 in the window region (6.09% above the best comparison)—demonstrating stronger local detail retention under challenging low-light, high-contrast conditions. Taken together, these results confirm that our approach excels in both visual fidelity and quantitative performance.

Overall, while GFF can excel in scenes with relatively stable lighting (e.g., daytime conditions), it can fall short in extreme low-light or high-contrast environments due to the strong gradient bias. Conversely, our method is specifically designed for microlight, high-dynamic-range scenarios, offering improved robustness against over-enhancement. At the same time, we acknowledge that no single approach is universally optimal, and each method may exhibit advantages or limitations depending on the ambient lighting and the desired balance between noise suppression and detail preservation.

### 4.5. Comparison of Operating Speed

To further evaluate efficiency, we measured the runtime of each method on our local computing device at several time points. The partial results are summarized in Table 4, from which it can be seen that our approach ranks among the faster methods overall. However, in practical deployments—such as integrating the algorithm into FPGA-based microcontroller systems—the actual speed may vary significantly due to hardware-specific constraints. Consequently, the runtime figures in Table 4 reflect performance on our particular setup and should not be interpreted as exact indicators of real-world sensor or embedded implementations.

### 4.6. Discussion

A comprehensive analysis of the full-day results shows that GFF and Li17 pay more attention to gradient enhancement in the fusion process, and thus have higher detail sharpness in brighter scenes during the daytime, but are prone to halo or local blurring and distortion in low-light hours; Li20 improves the luminance of the low-light background more clearly and has certain deficiencies in detail clarity. By contrast, the method in this paper is able to maintain stronger detail retention and contrast control at night under low light by introducing a halo suppression strategy with dynamic weight calculation and guided filtering and is able to maintain the transition between light and dark naturally in the daytime scene, where it is not easy to produce overexposure or edge halos. Comparison of the metrics shows that, in terms of EN, AG, EI, SF, etc., the proposed method is in the leading or better position in multi-temporal fusion, which is especially suitable for low-light and high-dynamic-range environments; although SD and *Q_cb_* are not as high as some of the proposed methods at times, the overall visual balance and the adequacy of details in the dark part of the scene still emphasize the advantages of the proposed method. The method not only takes into account the better lighting conditions during the daytime, but also has more advantages in the low-light high-dynamic-range scenes at night, and sufficiently solves the problem where it is difficult to take into account the halo and detail enhancement in the fusion enhancement in the low-light high-dynamic-image scene, as well as the un-natural transition between light and dark in the scene during the daytime.

In conclusion, the method in this study has strong adaptability and robustness under both daytime and nighttime conditions and can maximize the details and levels while suppressing halos, which is suitable for full-time application scenarios with complex lighting variations, such as smart city security monitoring and nighttime imaging.

## 5. Conclusions

In this paper, an image enhancement method based on dynamic weight is proposed to solve the problem of industrial cameras that have difficulty in taking into account the details of both bright and dark areas in low-light scenes. By comprehensively considering the global average exposure, local brightness, and gradient information in the improved exposure function and introducing guided filtering to suppress halos in the weight calculation process, balance and enhancement in bright and dark regions under multiple lighting conditions are realized. Subsequently, the image is reconstructed layer by layer using pyramidal hierarchical fusion, which can both enhance the high-frequency details and effectively balance the overall brightness. A large number of experimental results show that the method described in this paper can achieve subjective visual effects and objective evaluation indexes superior to other contrast algorithms in the continuous light range from low to strong light. The method has outstanding performance in halo suppression and dark-texture retention, showing great potential for application in scenarios that require precise detail capture under complex and dynamic lighting conditions with lightweight hardware environments. The approach is not limited to the field of city security surveillance but can also be widely applied in areas such as intelligent transportation, urban monitoring, and remote sensing security, providing effective support for tasks like object recognition and detection.

We also acknowledge the growing impact of deep learning in image enhancement, especially for low-light tasks. While many neural-network-based approaches depend heavily on large-scale training datasets and advanced GPU resources, future work could explore lightweight neural architectures or hybrid strategies that merge data-driven learning with our dynamic weighting framework. This research direction may further elevate performance in low-light, high-dynamic-range imaging without compromising efficiency or deployability.

## Figures and Tables

**Table 1:** Evaluation index score of different methods at four time points when the lighting is better.

Time	Evaluation Index Scores for Each Methodology			
	GFF	Li17	Li20	Ours
7:00 a.m.	5.4784	5.2688	4.8475	5.5074
10:00 a.m.	5.5517	5.3004	4.6113	5.5144
1:00 p.m.	5.5451	5.2198	4.6056	5.3497
4:00 p.m.	5.5477	5.1049	4.8456	5.2937

**Table 2:** Data on evaluation index scores of different methods at four time points during the low-light hours.

Time	Evaluation Index Scores for Each Methodology			
	GFF	Li17	Li20	Ours
7:00 p.m.	4.9026	4.4214	4.4851	5.9993
10:00 p.m.	4.5658	4.04	5.1758	5.8570
1:00 a.m.	5.0077	4.9997	4.9859	5.7735
4:00 a.m.	5.3694	4.9487	4.5252	5.8834

**Table 3:** Histogram data analysis for 8:00 p.m. locally magnified sections.

Methodology	Wall and LED					Window				
	Gray-Level Standard Deviation	Peak Gray Level	Peak Frequency	Gray-Level Range	Information Entropy	Gray-Level Standard Deviation	Peak Gray Level	Peak Frequency	Gray-Level Range	Information Entropy
GFF	13,682	14,422	29	[9830; 65,484]	11.92	7625	13,576	20	[10,396; 50,510]	10.67
Li17	11,225	16,625	42	[13,659; 65,532]	11.57	5130	15,396	31	[12,068; 31,400]	9.84
Li20	8443	22,675	73	[21,687; 65,531]	11.10	7052	20,976	55	[14,560; 41,031]	9.48
Ours	13,572	15,110	20	[12,268; 65,523]	12.50	13,617	14,202	12	[6554; 54,379]	11.32

**Table 4:** The runtime of each method on our local computing device.

Methodology	Time (s)							
	7:00 a.m.	10:00 a.m.	1:00 p.m.	4:00 p.m.	7:00 p.m.	10:00 p.m.	1:00 a.m.	4:00 a.m.
GFF	27.73	17.17	17.04	20.11	43.99	31.92	31.99	32.17
Li17	56.82	49.61	50.49	58.15	68.16	67.39	67.78	67.05
Li20	16.11	14.15	14.27	14.63	40.88	27.09	28.52	28.32
Ours	18.97	12.60	13.78	15.00	41.05	30.38	31.78	31.84

## Data Availability

Data are contained within the article.
